# Arabidopsis NUCLEOSTEMIN-LIKE 1 (NSN1) regulates cell cycling potentially by cooperating with nucleosome assembly protein AtNAP1;1

**DOI:** 10.1186/s12870-018-1289-2

**Published:** 2018-06-01

**Authors:** Zhen Wang, Xiaomin Wang, Bo Xie, Zonglie Hong, Qingchuan Yang

**Affiliations:** 1 Institute of Animal Science, Chinese Academy of Agricultural Sciences, Beijing, 100193 China; 2 Department of Plant Sciences, University of Idaho, Moscow, Idaho 83844 USA

**Keywords:** Cell cycle, Cell proliferation, Nucleostemin-like1, Nucleosome assembly protein1

## Abstract

**Background:**

In mammals, nucleostemin (NS), a nucleolar GTPase, is involved in stem cell proliferation, embryogenesis and ribosome biogenesis. Arabidopsis *NUCLEOSTEMIN-LIKE 1* (*NSN1*) has previously been shown to be essential for plant growth and development. However, the role of NSN1 in cell proliferation is largely unknown.

**Results:**

Using *nsn1*, a loss-of-function mutant of Arabidopsis *NSN1*, we investigated the function of NSN1 in plant cell proliferation and cell cycle regulation. Morphologically, *nsn1* exhibited developmental defects in both leaves and roots, producing severely reduced vegetative organs with a much smaller number of cells than those in the wild type. Dynamic analysis of leaf and root growth revealed a lower cell proliferation rate and slower cell division in *nsn1*. Consistently, the transcriptional levels of key cell  cycle genes, including those regulating the transition of G1-S and G2-M, were reduced drastically in *nsn1*. The introduction of *CYCLIN B1::GUS* into *nsn1* resulted in confined expression of GUS in both the leaf primordia and root meristem, indicating that cell proliferation was hampered by the mutation of *NSN1*. Upon subjection to treatment with bleomycin and methyl methanesulfonate (MMS), *nsn1* plants exhibited hypersensitivity to the genotoxic agents. In the nucleus, NSN1 interacted with nucleosome assembly protein1 (AtNAP1;1), a highly conserved histone chaperone functioning in cell proliferation. Notably, the N-terminal conserved domains of Arabidopsis NSN1 were critical for the physical interaction.

**Conclusions:**

As a conserved homolog of mammalian nucleostemin, Arabidopsis NSN1 plays pivotal roles in embryogenesis and ribosome biogenesis. In this study, *NSN1* was found to function as a positive regulator in cell cycle progression. The interaction between NSN1 and histone chaperone AtNAP1;1, and the high resemblance in sensitivity to genotoxics between *nsn1* and *atnap1;1* imply the indispensability of the two nuclear proteins for cell cycle regulation. This work provides an insight into the delicate control of cell proliferation through the cooperation of a GTP-binding protein with a nucleosome assembly/disassembly protein in Arabidopsis.

**Electronic supplementary material:**

The online version of this article (10.1186/s12870-018-1289-2) contains supplementary material, which is available to authorized users.

## Background

In multicellular organisms, organogenesis requires tight control of cell proliferation. Nucleostemin (NS), a nucleolar GTP-binding protein, was first identified in stem cells and some cancer cell lines in mouse [1]. It has been implicated in stem cell proliferation, cell cycle maintenance, ribosome biogenesis and embryogenesis [1–4]. For example, depletion or overexpression of NS in mouse caused arrest of G1-S and G2-M transition by inhibiting the activity of Murine Double Minute 2 (MDM2), an E3 ubiquitin ligase targeting p53 for proteasome-mediated degradation, from activating p53 [1, 3, 5, 6]. NS was found in a large protein complex containing pre-rRNA processing related nucleolar proteins, such as Pescadillo (Pes1), DDX21 (also known as RHII/Guα) and EBNA1 binding protein 2 (EBP2) [7], suggesting a key role in ribosome biogenesis.

As a member of the conserved GTPases present in prokaryotes and eukaryotes, NS belongs to the YlqF/YawG GTPase family [8, 9]. The human NS family consists of nucleostemin, Guanine Nucleotide binding protein-like 3 (GNL3L) and Ngp-1, and all the three members have been shown experimentally to bind GTP in vivo, which is consistent with the presence of the GTP binding domain in these proteins [3, 10, 11]. In addition, NS also contains an acidic amino acid (AAA) domain at the C-terminus, and a basic amino acid (BAA) domain and a coiled-coil (CC) domain at the N-terminus [1]. The latter two domains contribute predominantly to its biological functions as a nucleolar protein in cell cycle progression of both animals and plants [1, 7, 12–14].

Unlike in mammals, only two subfamilies of NS genes (*nucleostemin* and *Ngp-1-like*) have been identified in Arabidopsis with the absence of *GNL3L-like* family genes [15]. Nucleostemin-like1 (NSN1) of Arabidopsis has been  found to posess the common domains of mamalian NS and GFP-fused NSN1 has been  mainly localized to the nucleolus in tobacco BY-2 cells [15]. The *nsn1* mutant exhibits defects in embryogenesis and in the development of both leaf and flower organs. Consistently, *NSN1* is highly expressed in the developing embryos, floral and shoot apical meristems, and organ primordia [15, 16]. The observation of reduced expression of meristem genes including *WUS*, *CLV3, STM* and *ANT* in *nsn1* suggests that *NSN1* plays critical roles in embryogenesis [15]. Recently, a role of NSN1 in plant growth and senescence by modulating ribosome biogenesis has been reported based on the observations that silencing *NSN1* leads to growth retardation and premature senescence in Arabidopsis and tobacco [14]. Several ribosomal proteins including Pescadillo and EBP2 have been found to interact with NSN1 and depletion of NSN1 has been shown to repress global translation probably by delaying the biogenesis of the 60S ribosome subunit [14]. Taken together, Arabidopsis NSN1 plays important roles in plant growth and development although the exact mechanism is not clear.

NUCLEOSOME ASSEMBLY PROTEINS1 (NAP1), a histone chaperone in nucleosome assembly/disassembly, is highly conserved from yeast to human [17–20]. NAP1 deficiency is known to cause perturbed expression of around 10% of nuclear genes in yeast [17, 20], and embryonic lethality in mammals and fruit fly [20, 21]. In plants, *NAP1* has been identified in several species including rice, soybean, tobacco and Arabidopsis [18, 19, 22, 23]. In Arabidopsis, *NAP1* is a multi-gene family containing 4 members: *AtNAP1;1*, *AtNAP1;2*, *AtNAP1;3* and *AtNAP1;4*. Phenotypically the individual mutant resembles wild type plants under normal growth conditions, suggesting the functional redundance of the paralogs [18, 24]. Two alleles (*m123-1* and *m123-2*) of the triple mutant *Atnap1;1 Atnap1;2 Atnap1;3* exhibit hypersensitivity to DNA damage caused by UV-C irradiation [18], revealing a role of *AtNAP1* genes in nucleotide excision repair of DNA. Research on AtNAP1;1 has revealed that like its orthologs in rice and tobacco, some AtNAP1;1-GFP fusion proteins are targeted to  the nucleus at an early stage of leaf development whereas the abundance of the fusion proteins are retained  in the cytoplasm [22]. Its function in promoting cell division or expansion and the farnesylation status of the protein has been found to be coupled with its subcellular localization [24]. In a bimolecular fluorescence complementation (BiFC) assay, as well as a pull-down experiment, AtNAP1;1 has been demonstrated to interact with ribosomal protein S6 (RPS6), supporting its positive regulatory role in plant rDNA transcription [25]. Analysis of a truncated protein of AtNAP1;3, which lacks 34 amino acids at the C-terminus, reveals altered Arabidopsis responses to abscisic acid (ABA) treatment and salt stress by functioning as a dominant negative factor in ABA responses [23].

In addition to NAP1, Arabidopsis has two NAP1-related proteins, AtNRP1 and AtNRP2. Both are primarily localized in the nucleus, and the double mutant *Atnrp1-1Atnrp2-1* exhibits short roots and increased sensitivity to the genotoxic treatment [2]. A recent study using reporter constructs of homologous recombination (HR) has demonstrated reduced levels of HR in the double mutant *Atnrp1-1Atnrp2-1* [26] and the *AtNAP1* triple mutant *m123-1,* suggesting that NRP and AtNAP1 act in parallel pathways to synergistically promote somatic HR.

In this study, we focused on the function of *NSN1* in cell cycle regulation. Molecular, histochemical and genetic analysis demonstrated that the growth retardation of dwarf mutant *nsn1* is attributed to its arrested cell cycle. The interplay between NSN1 and AtNAP1;1, their involvement in cell cycling progression and the hypersensitivity of *nsn1* and *atnap1;1* to the genotoxic agents imply that in Arabidopsis *NSN1* regulates cell cycling and DNA damage repair by cooperating with histone chaperone protein AtNAP1;1.

## Results

### Growth defects of *nsn1* mutant

**Fig. 1:**
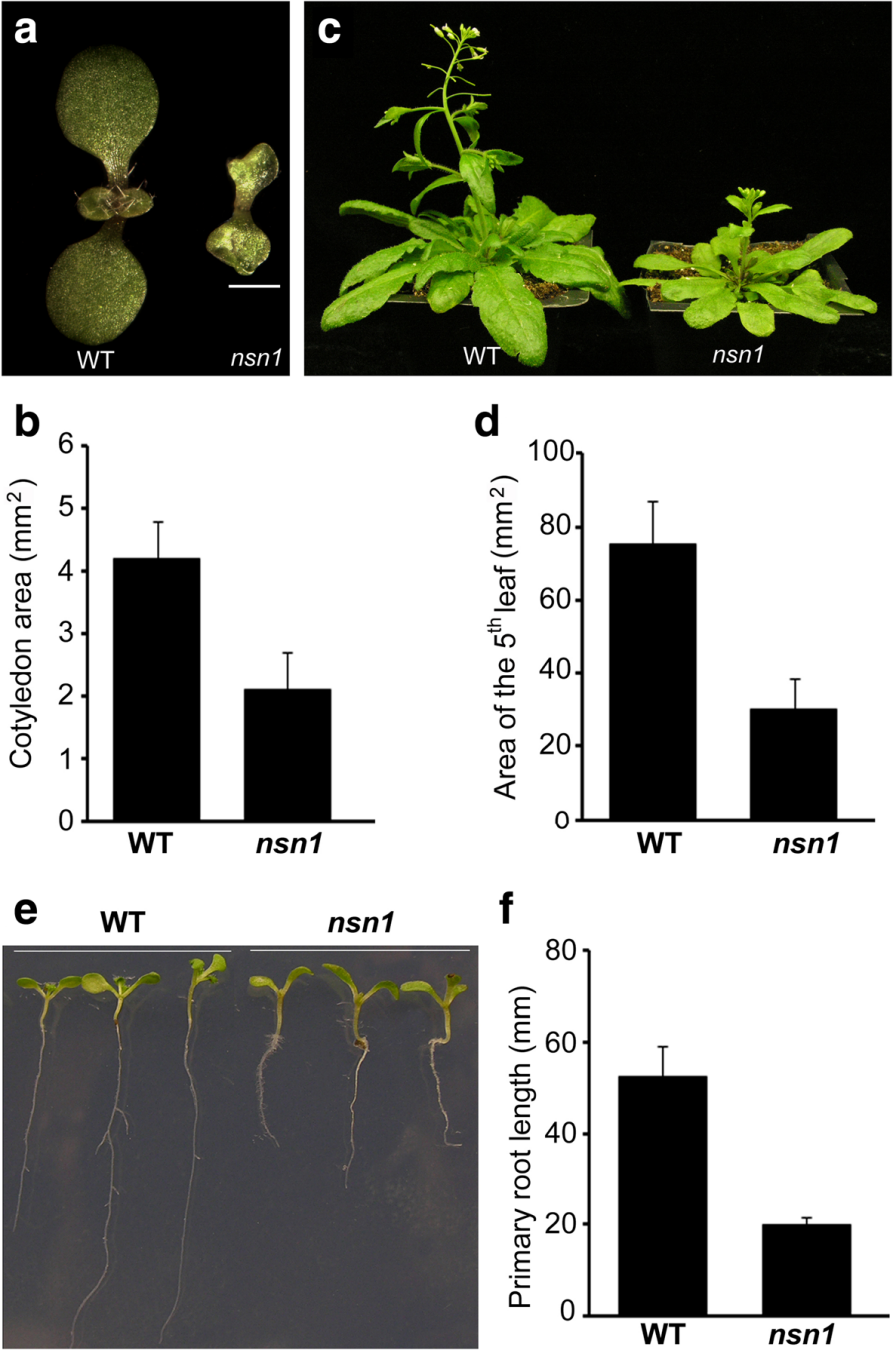
Retarded growth of leaf and root organs in *nsn1*. **a** Eight-day-old seedlings of wild type and *nsn1*, showing reduced size of cotyledons and late initiation of the first leaf in *nsn1.* Scale bar: 1 mm. **b** Statistical analysis of cotyledon area of 8-day-old seedlings. **c** Twenty-one-day-old wild type and *nsn1* plants. **d** Leaf area analysis of the fifth leaf from 21-day-old plants. **e** Ten-day-old seedlings grown on MS medium showing retarded root growth in *nsn1* as compared with the wild type. **f** Statistical analysis of the primary root length of 10-day-old seedlings. For the statistical analysis, three biological replicates were conducted with 15 plants measured for each experiment

In previous studies, *Nucleostemin-like1* (*NSN1*) in Arabidopsis has been demonstrated to be essential for the growth of both vegetative and reproductive organs by participation in regulation of embryogenesis and meristem development [14–16]. Here, we investigated the role of *NSN1* in cell proliferation and division in both leaf and root organs. Consistent with the findings that cotyledons of *nsn1,* a loss-of-function mutant of *NSN1*, are initiated late at the embryo stage [15, 16], delayed emergence of the first leaf in *nsn1* dwarf plant was observed (Fig. 1a) with the first leaf emerging at 9th day after germination (DAG), compared with the 5th DAG in the wild type plants (Table 1). For the duration of leaf expansion, at the 9th day after initiation (DAI), the fifth leaf of wild type reached maximum leaf area, while the counterpart leaf of *nsn1* fully expended and achieved the maximum area at the 21st DAI (Table 1). Therefore, *nsn1* displayed severe growth retardation during the life cycle. The fifth leaf pair was used in this study unless otherwise indicated. Analysis of the leaf area revealed that the size of cotyledons and the fifth leaf of *nsn1* were about 50 and 40% of their counterparts in wild type, respectively (Fig. 1b, d). As a result, *nsn1* with smaller size of leaves exhibited dwarfism (Fig. 1c). Similar developmental retardation was observed in roots of *nsn1* plants, whose primary root length was about 40% of that of wild type (Fig. 1e, f). Therefore, both aerial and underground organs of *nsn1* plants displayed growth defects, suggesting the indispensability of *NSN1* for plant growth.

**Table 1:** Comparison of leaf development between wild type and *nsn1*

	Emergence of 1st. leaf (DAG)	Full expansion of 5th. leaf (DAI)
Wild type	5.1 ± 1.2	9.3 ± 1.8
*nsn1*	9.5 ± 1.9	21.2 ± 2.4

Note: *DAG* days after germination; *DAI* days after initiation. Value represents mean ± SD. Three biological replicates were performed with at least 15 plants measured for each experiment

### Deficient cell proliferation in *nsn1* leaf

**Fig. 2:**
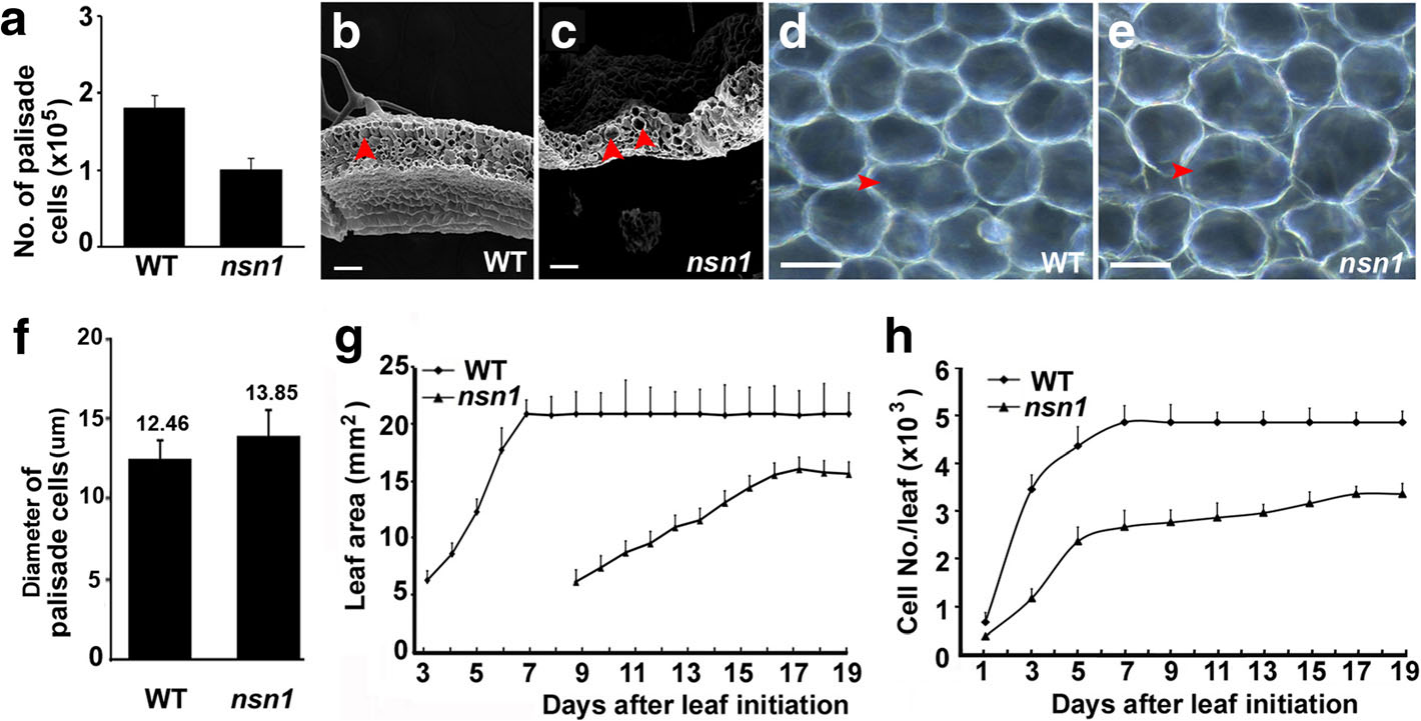
Inefficient cell proliferation in *nsn1* leaves. The fifth leaf pair was used for measurement and analysis. **a** Statistical analysis of the number of palisade cells from fully expanded leaves. Scanning electron microscope (SEM) images of fully expanded leaves of wild type (**b**) and *nsn1 *(**c**). Arrow heads indicate the palisade cells with similar size from the two genotypes. The palisade cells of fully expanded leaves of wild type (**d**) and *nsn1* (**e**). **f** Statistical analysis of the diameter of palisade cells from fully expanded leaves. **g** Dynamics of leaf expansion of wild type and *nsn1*. **h** Dynamic analysis of cell number/leaf of wild type and *nsn1*. The difference between the two datasets was analyzed by Student’s t test (*P* < 0.01). Scale bars: 50 μm for (**b)** and (**c**); 10 μm for (**d)** and (**e**)

Plant organ size is genetically determined by both the number and the size of its constituent cells. To investigate the cause of dwarfism in *nsn1*, the number and size of palisade cells of the fifth leaf pair were measured and analyzed. In agreement with the dwarf statue of *nsn1*, the amount of the palisade cells was reduced to 56% of that of the wild type (Fig. 2a). In terms of cell size, although cells in the palisade cell layer from the cross-section of *nsn1* leaves were slightly larger than those of the wild type (Fig. 2b-e), statistically the diameter difference of the palisade cells between the two genotypes was not significant (*P* > 0.05) (Fig. 2f), suggesting that the cell size is not the major source for the dwarfism in *nsn1*. Hence, the reduction of the overall leaf size of *nsn1* mutant is attributed to the decrease of cell numbers rather than cell size alteration.

To search for the cause of cell reduction in *nsn1*, dynamics of leaf area and cell proliferation rate were examined and compared with the wild type plants. Kinetic analysis of leave growth showed that the leaf area of wild type increased dramatically after initiation and reached the maximum at 7th DAI, while the progressive leaf growth of *nsn1* was relatively slow and the maximum value was achieved at 17th DAI (Fig. 2g). The long duration of cell proliferation in *nsn1* indicated a slower cell proliferation rate in the dwarf mutant. Synchronically, dynamics of cell number/leaf demonstrated a significant difference of the maximum cell number between *nsn1* and wild type, with about 3.4 × 10^3^ at 17th DAI for *nsn1* and 4.9 × 10^3^ cells at 7th DAI for wild type (Fig. 2h), respectively. Taken together, the hampered leaf growth in *nsn1* was resulted from the malfunction of *NSN1* in cell proliferation. Additionally, in wild type the palisade cell layer and spongy cell layer are easily differentiated and the longitudinal axial of elongated palisade cell is vertical to the leaf epidermis (Fig. 2b). In *nsn1*, however, abnormal palisade cells with irregular patterning and highly variable size and shape were observed (Fig. 2c), suggesting that besides regulating cell proliferation and division, *NSN1* may function in leaf development as well.

### Inefficient cell proliferation in *nsn1* roots

**Fig. 3:**
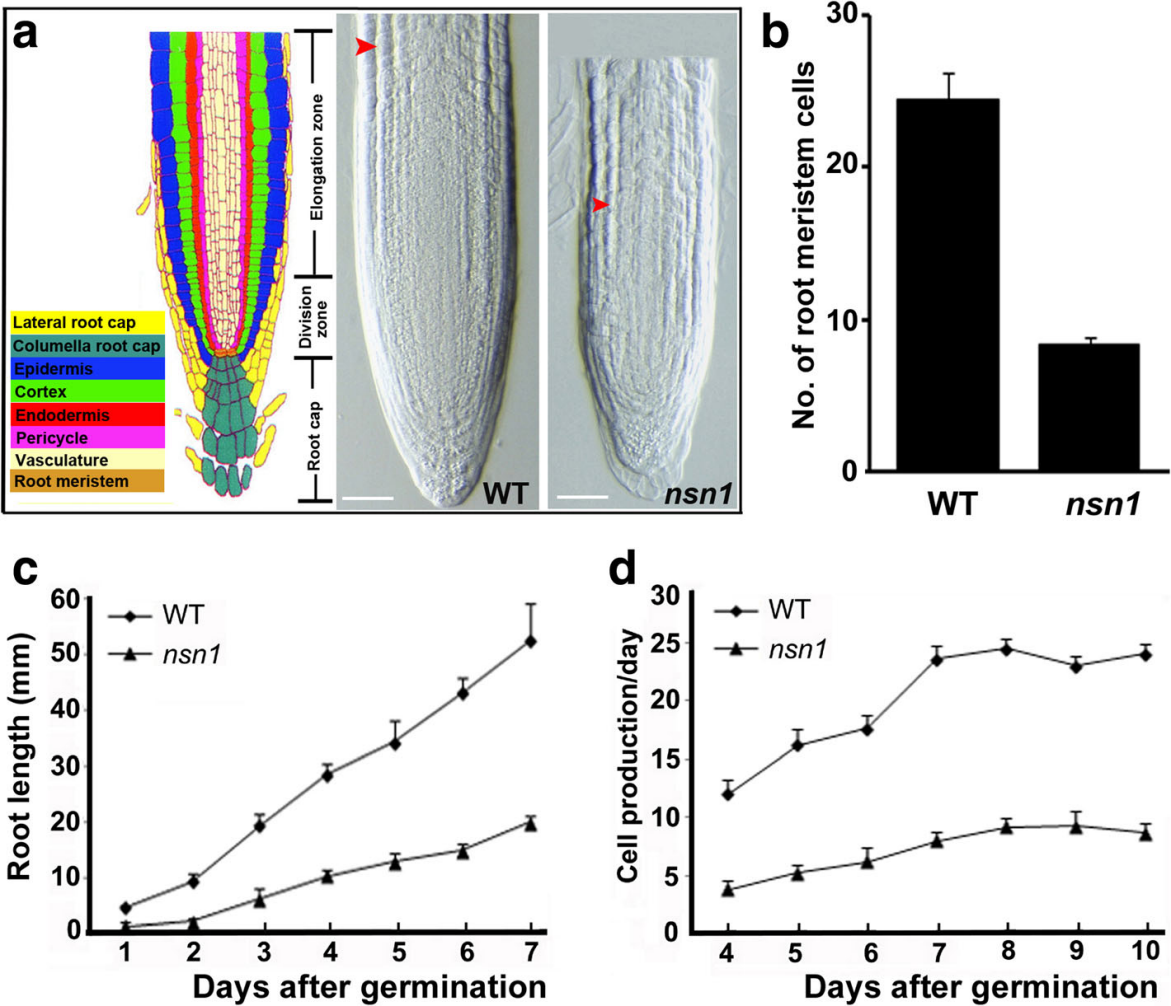
Inefficient cell proliferation in *nsn1* root. **a** Longitudinal sections of the primary root from 10-day-old wild type and *nsn1*. Illustration of the tissue organization of the Arabidopsis primary root apex (left in color) was adapted from [42]. Arrowhead indicated the cortex cells in elongation zone. Scale bar: 100 μm. **b** Statistical analysis of cell numbers in the root meristem region of 10-day-old plants. **c** Comparison of the dynamics of the primary root length in wild type and *nsn1* within the first week after germination. **d** Analysis of the daily cell production rate in the primary root of wild type and *nsn1*. The number of cortex cells at the root tip including elongation and cell division zones was counted under microscope daily from the 4th day to the 10th day after germination. Cortex cells on one side as indicated by arrowheads in (**a**) were calculated

In agreement with the retardation of root growth in *nsn1* (Fig. 1e, f), 10-day-old mutant exhibited shorter primary roots and a smaller number of meristematic cells were present in the root meristem region of *nsn1* than in wild type (Fig. 3a, b). In terms of root growth rate, root length was monitored during the first week after germination. The length of primary root of *nsn1* was about 25–35% of wild type within the first 7 days (Fig. 3c), and on day 10, *nsn1* root reached 40% of wild type (Fig. 1e, f). Consistent with the measurement of meristem cells, the number of cells produced by *nsn1* roots per day was about one third of that of the wild type roots (Fig. 3d). Therefore, our analysis indicates that slower root elongation and less daily root cell production of *nsn1* account for the observed growth defects in the mutant.

### Impaired cell cycling progression in *nsn1*

**Fig. 4:**
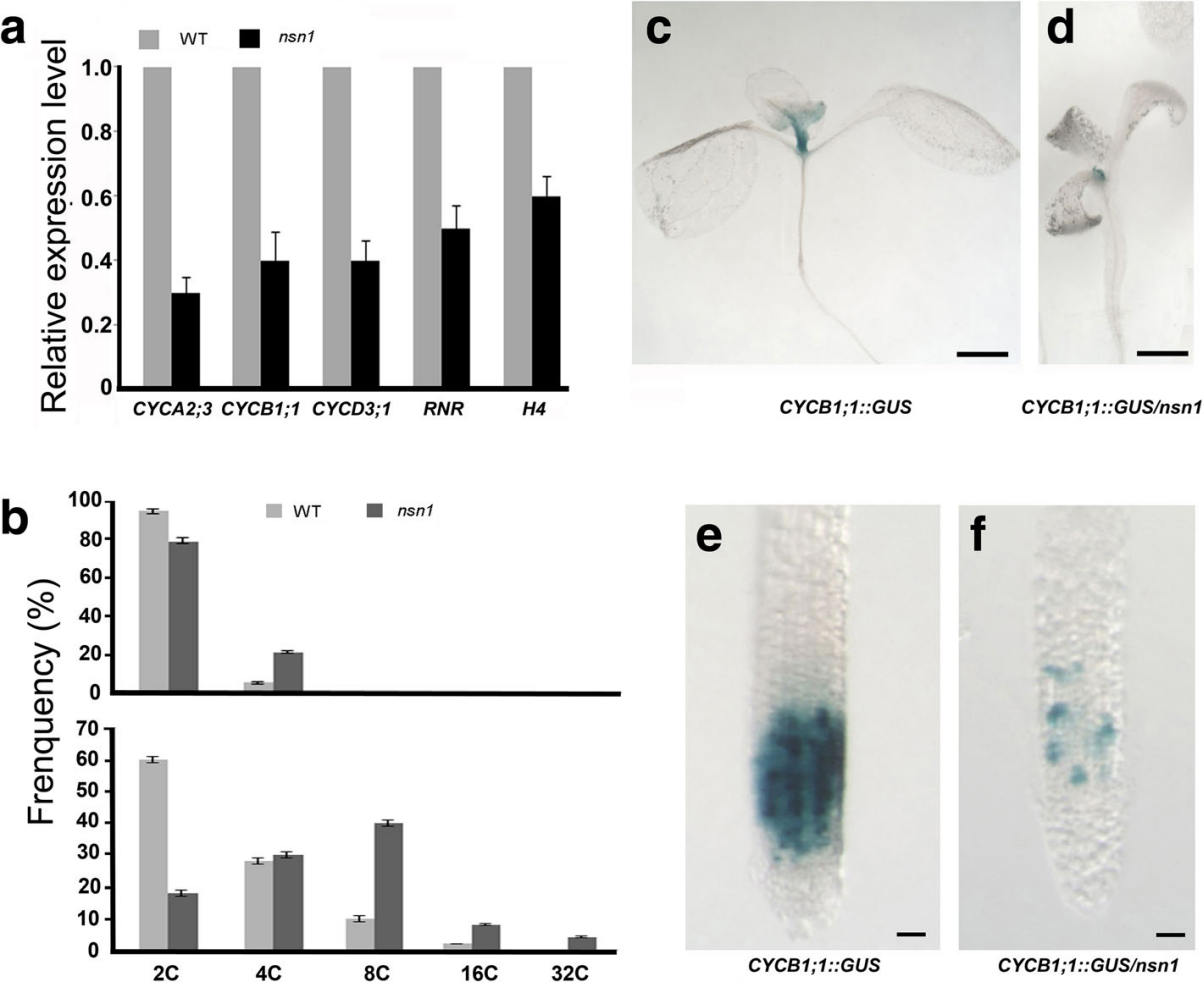
Analysis of cell cycle defects in *nsn1*. **a** Relative transcriptional levels of cell cycle genes in *nsn1* as measured by q-RT-PCR. Ten-day-old seedlings shown in Fig. 1e were used for total RNA extraction after the root length measurement. Three biological replicates were conducted with 15 plants for each experiment. **b** Flow cytometry assay of nuclei isolated from leaves of 4-days after initiation (upper panel) and of fully-expanded fifth leaves (lower panel). **c**-**f** Histochemical analysis of GUS signal in ten-day-old seedlings of indicated genotypes. GUS signal in the shoot apical meristem (**c**) and root meristem (**e**) of *CYCB1;1::GUS* expressing wild type plants stained with X-glux solution. GUS signal in the shoot apical meristem (**d**) and root meristem (**f**) of *CYCB1;1::GUS* expressing *nsn1* plants stained with X-glux solution. Scale bars: 0.5 mm for (**c**) and (**d**); 100 μm for (**e**) and (**f**)

Given that the dwarfism of *nsn1* was resulted from defective cell proliferation and division, we investigated whether the *nsn1 *mutation affected the transcriptional levels of five cell cycle marker genes, including three cyclin family genes *CYCA2;3*, *CYCB1;1,* and *CYCD3;1*, and two S-phase specific genes *HISTONE H4* and *Ribonucleotide reductase* (*RNR)* [27]. For the three cyclin genes, *CYCD3;1* has been reported as a key gene in G1-S transition by controlling cell division rate [28], while the remaining two genes, *CYCB1;1* and *CYCA2;3,* have been found to affect G2/M transition in cell cycle [29, 30]. Quantitative analysis revealed that in *nsn1*, the expression level of the cyclin genes was about 30–40% of that in wild type, and the transcriptional level of *HISTONE H4* and *RNR*, a key enzyme in the DNA synthesis pathway [27], decreased to approximately 60 and 50% of those in wild type, respectively (Fig. 4a). The notable down-regulation of these cell cycle regulating genes in *nsn1* implied the disruption of both G1-S and G2-M transition.

To further dissect the involvement of NSN1 in cell cycle regulation, flow cytometry analysis was performed. Leaves from two developmental stages (4-day-old and mature) were subjected to nuclei isolation. Our results showed a lower percentage of 2C nuclei in *nsn1* than wild type at both the young and mature phases: 79 vs. 95% and 18 vs. 60%, respectively (Fig. 4b). Consistently, compared with the wild type, in *nsn1*, 16% more of 4C nuclei were detected in young leaves, and 42% more of polyploidy including 4C, 8C, 16C and 32C nuclei were observed in mature leaves (Fig. 4b). The detection of higher percentage of polyploidy indicated a condensed DNA content in *nsn1* due to the malfunction of *nsn1* mutation in cell division.

To visualize the effect of *NSN1* on cell cycle progression, transgenic plants expressing *CYCLIN* *B1;1::GUS*, which expresses the reporter β-glucuronidase (GUS) fused to the mitotic destruction sequence (D-box) under the promoter of the cyclin *CYCB1;1* [31], were crossed with *nsn1* mutant to obtain *CYCLIN* *B1;1::GUS*-containing *nsn1* lines. The GUS activity marked cells in G2 and early M phase [31] because the fusion gene was expressed upon entry into G2 (via the *CYCB1:1* promoter) and its protein product was degraded upon exit from metaphase (via D-box) [32]. Histochemical analysis showed that in the wild type background, GUS-positive cells were predominantly detected in the regions of leaf primordia (Fig. 4c). When *CYCLIN* *B1;1::GUS* was introduced into *nsn1*, however, the expression of GUS was confined to a much smaller area containing meristematic cells of the shoot apical region (Fig. 4d). In roots, a clearly lower number of GUS-positive cells were observed in *nsn1* plants than those in the control plant (Fig. 4e, f). The restricted *CYCLIN* *B1;1::GUS* signal in both the root and shoot apical meristems of *nsn1* plants provided genetic evidence of arrested cell cycling in *nsn1*. Therefore, our molecular and genetic data revealed that the cell cycle progression in both leaves and roots of *nsn1* was impaired by the *nsn1* mutation.

### Hypersensitivity of *nsn1* to genotoxic agents

**Fig. 5:**
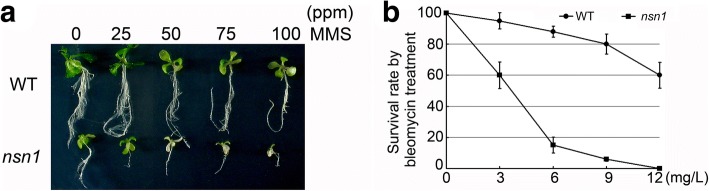
Comparison of sensitivity to genotoxic agent treatment. **a** Four-day-old seedlings were treated with MMS at the indicated concentrations for 1 week. **b** Four-day-old seedlings were treated with bleomycin at the different concentrations for 3 weeks. Three biological replicates were performed with at least 15 plants tested per experiment

Given that *NSN1* is essential for proper cell cycle progression, plant sensitivity to DNA damaging agents methyl methanesulfonate (MMS) and bleomycin was tested. MMS is known to result in alkylated DNA, which is poorly replicated by DNA polymerases [33], while treatment with bleomycin, a radiomimetic drug, causes multiple types of molecular damages including double strand breaks (DSBs) [34]. Four-day-old Arabidopsis plantlets were subjected to treatment with MMS and bleomycin. When *nsn1* seedlings were exposed to 25 ppm MMS, rosette leaves turned yellow and root growth was severely inhibited (Fig. 5a). MMS of 50 ppm inhibited the growth of leaf and root organs, resulting in leaf etiolation and plant death. The damages caused by MMS was dosage-dependent (Fig. 5a). In contrast, the growth of wild type was not obviously affected by MMS at 25 ppm, and for treatment of 100 ppm MMS, no plant death was observed although rosette leaves were yellowish and root growth was badly inhibited (Fig. 5a). These results indicated that *nsn1* is hypersensitive to MMS treatment in comparison with the wild type. The survival rate of *nsn1* plants was dramatically decreased by bleomycin treatment, especially at dosages higher than 6 mg/L (Fig. 5b). At the dosage of 12 mg/L, all *nsn1* plantlets died after being treated for 1 week, while about 60% of wild type plants survived the treatment (Fig. 5b). Hence, *nsn1* displayed more pronounced sensitivity to the two genotoxic agents than the wild type plants, implying the involvement of NSN1 in cell cycle progression in plants.

### Interaction of NSN1 with AtNAP1;1 in vitro and in vivo

Like mammalian NS, Arabidopsis NSN1 interacts with several nucleolar proteins involved in ribosomal biogenesis [14]. To search for potential plant-specific protein(s) physically interacting with NSN1, a yeast two-hybrid screen was carried out. We screened a cDNA library of Arabidopsis flower from ABRC (stock CD4-30) constructed in pAD-GAL4-2.1. *NSN1* cDNA was cloned into pBD-GAL4 (Strategy, USA) and served  as bait. Empty vectors of pBD-GAL4 and pAD-GAL4-2.1 were used as negative controls for verification of candidate clones.

**Fig. 6:**
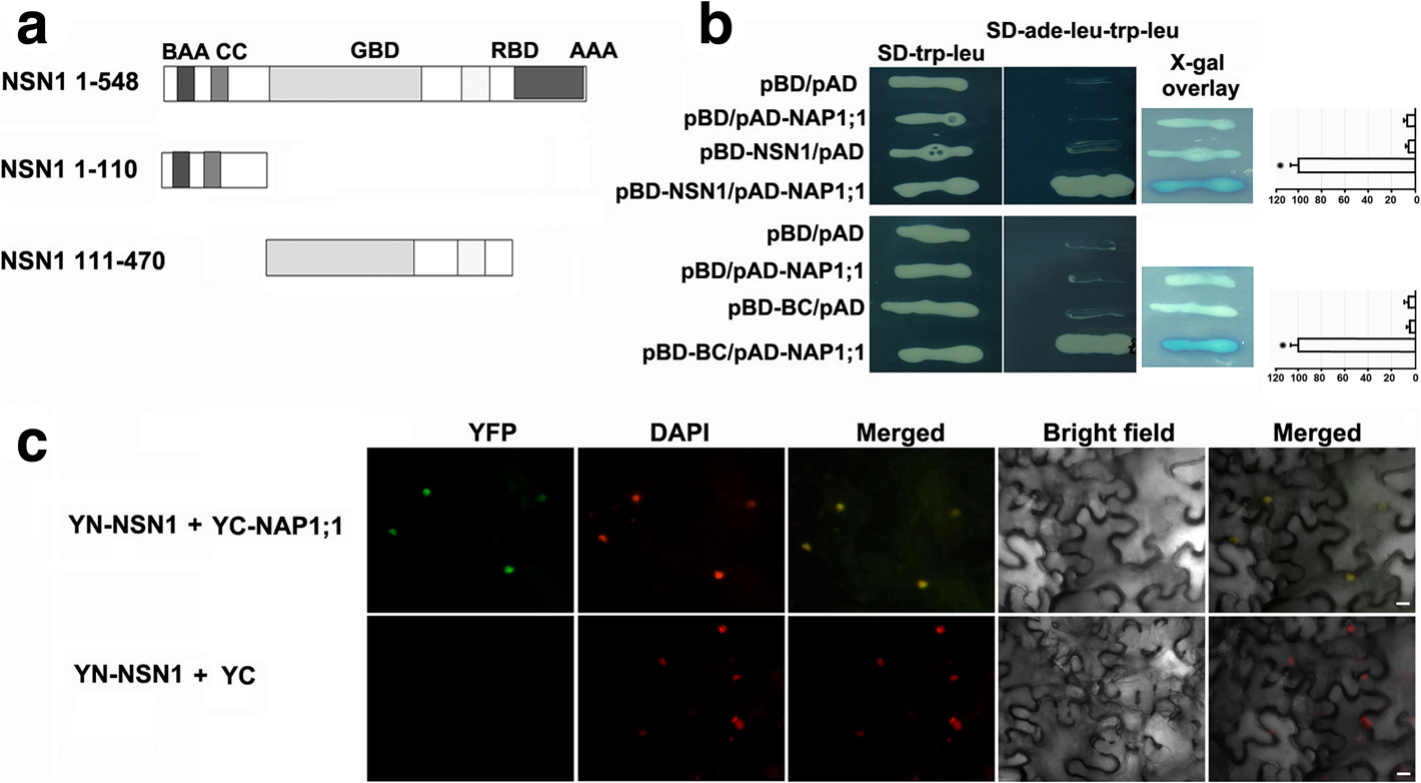
Interaction between NSN1 and NAP1;1 in yeast and tobacco. **a** Illustration of NSN1 fragments used for yeast two-hybrid assays. NSN1 protein consists of 548 amino acids. Two fragments tested in the yeast two-hybrid assay are the N-terminal fragment (amino acids 1–110) including the basic amino acid (BAA) domain and coiled-coil (CC) domain, and the fragment of amino acids 111–470 representing the GTP binding domain (GBD) and RNA binding domain (RBD). **b** Analysis of in vitro interaction between NAP1;1 and NSN1 with or without a domain deletion using the yeast two-hybrid system. The full length *NSN1* was cloned into pBD vector as bait to screen a library of flower from ABRC (upper panel). A positive interaction colony was confirmed to contain At*NAP1;1* (At4g26110.2) by DNA sequencing. pAD-NAP1;1 represents the full length CDS of *AtNAP1;1* cloned into pAD vector. The upper panel displays the interaction between AtNAP1;1 and NSN1; The lower panel shows the interactions between AtNAP1;1 and the N-terminal fragment of NSN1 including the basic amino acid (BAA) domain and coiled-coil (CC) domain, which was abbreviated as BC. Empty vectors pBD and pAD were used as negative controls. X-gal overlay assays and the quantification of ß-galactosidase activity were shown (right panels). Significant difference (*p* < 0.001) was evaluated by Student’s t test. **c** Analysis of in vivo interaction between NSN1 and NAP1;1 using biomolecule fluorescence complementation (BiFC) in tobacco leaves. An enhanced GFP (eGFP) was used [35] to test the interaction by fusing the coding sequence of *NSN1* and *AtNAP1;1* with the N-terminal (YFP^N^-NSN1) and the C-terminal fragment of GFP (YFP^C^-NAP1;1), respectively. The two constructs were co-expressed in tobacco leaves by *Agrobacterium*-infiltration. Infiltrated leaf epidermal cells were checked with confocal laser scanning. The co-expression of YFP^N^-NSN1 and no-fusion pSPYCE (M) 155 (YC) was used as a negative control. Nuclei were stained with DAPI. Scale bars: 10 μm

Around a million yeast colonies were screened and candidate colonies were verified to eliminate false positives. One colony showed consistent growth on the selective media, and the colony was identified as *NAP1;1 *(At4g26110.2), a member of the NUCLEOSOME ASSEMBLY PROTEINS1 (NAP1) [24]. Further analysis demonstrated that the yeast colony expressing the full length coding sequence (CDS) of *AtNAP1;1* grew healthily on the selective media and turned blue on X-gal indicator plates (Fig. 6b), suggesting an interaction between the encoded protein and NSN1. For the control vectors, as expected, yeast hardly grew on the selective media with extremely faint blue in X-gal overlay assay (Fig. 6b upper panel). Quantification of the interaction strength revealed a significantly strong signal in the cells expressing both NSN1 and AtNAP1;1 (Fig. 6b upper panel), indicating that the presence of both NSN1 and AtNAP1;1 is required for the observed interaction. Interestingly, similar to *NSN1*, *AtNAP1;1* has also been demonstrated to regulate cell proliferation and leaf development [24].

NSN1 contains the common conserved domains of nucleostemin, including the basic amino acid domain (BAA), coiled-coil domain (CC), GTP-binding domain (GBD), RNA binding domain (RBD) and acidic amino acid domain (AAA) (Fig. 6a). To investigate which domains of NSN1 were indispensable for the in vitro interaction with AtNAP1;1, two adjacent combined domains, including BC standing for the N-terminal BAA and CC domains, and GR for the GBD and RBD domains, were tested (Fig. 6a). Domain deletion analysis demonstrated that with the BC domains, yeast growth and strong blue signal were observed, while the controls comprising either of the tested peptides with an empty vector were negative (Fig. 6b), suggesting that the presence of the two N-terminal domains, BAA and CC, of NSN1 is essential for the interaction (Fig. 6b). No physical interaction was detected between AtNAP1;1 and the two middle domains of NSN1, e.g. GBD and RBD (data not shown). These results indicated that the N-terminal two conserved domains (BAA and CC), but not the middle two domains (RBD and AAA), were essential for the physical interaction between NSN1 and AtNAP1;1.

To test whether NSN1 and AtNAP1;1 interact in vivo, the BiFC assay, a widely used tool for visualization of protein-protein interaction in living cells, was performed. The coding sequences of NSN1 and AtNAP1;1 were fused in frame with the N-terminal and C-terminal fragments of enhanced GFP (eGFP), respectively [35]. The fusion proteins (YFP^N^-NSN1 and YFP^C^-NAP1;1) were co-expressed in tobacco leaves by *Agrobacterium*-infiltration. Leaf epidermal cells of infiltrated tobacco were checked with confocal laser scanning microscopy after 48 h. GFP fluorescence was observed in the nuclei of tobacco leaves transfected with YFP^N^-NSN1 and YFP^C^-NAP1;1 as indicated by DAPI-stained nuclei (Fig. 6c). This result is consistent with the previous studies that NSN1 was predominantly localized in nucleoli [14, 16]. In contrast, no YFP signal was detected when YFP^N^-NSN1 was co-expressed with no-fusion pSPYCE (M) 155 (YC), as the control, in tobacco cells (Fig. 6c). These results demonstrated that NSN1 interacted in vivo with AtNAP1;1, one of AtNAP1 proteins functioning as a histone chaperone in nucleosome assembly/disassembly.

## Discussion

Plant Nucleostemin homologs have been identified in recent years from Arabidopsis and tobacco [14, 15]. It has been reported that Arabidopsis *NSN1* plays critical roles in floral meristem development, floral organ identity, embryogenesis, leaf development and senescence [14–16]. The aim of this work was to functionally characterize *NSN1* in cell cycle regulation in Arabidopsis. Based on the molecular and genetic analysis of the cell cycling components and the dynamics of cell proliferation in *nsn1* mutant, we concluded that *NSN1* is indispensable for correct cell proliferation control. The identification of AtNAP1;1, a histone chaperone, as an interacting partner of NSN1 implies that the two proteins might function in a nucleolar complex to regulate cell cycle progression.

Arabidopsis *NSN1* is involved in plant growth and development by maintaining proper cell cycle progression. It has been documented that in animals when *NS* is deleted or overexpressed, cell cycling is arrested and the transition of G1-S and G2-M is stopped [3, 5, 6]. Given that the Arabidopsis loss-of-function mutant *nsn1* exhibits dwarfism and NSN1 is localized primarily in the nucleolus where the key features of cell growth occur, *NSN1* may affect plant cell cycle progression. This view is supported by the molecular evidence of a uniform down-regulation of core cell cycling genes, such as *CYCA2;3*, *CYCB1;1, CYCD3;1*, *HISTONE H4* and *RNR*, in *nsn1* (Fig. 4a). In addition to controlling cell-cycle transition, the core cell cycling components have been documented to function in coordinating cell division with differentiation and development in plants [36, 37]. The observations of higher percentages of polyploidy, severely reduced numbers of CYCLIN B1;1::GUS-expressing cells and slower cell proliferation in *nsn1* suggest the deficiency in cell division. Consequently, growth retardation was exhibited in both leaves and roots of nsn1, and has also been observed in *NbNSN1* silencing tobacco plants as well [14]. These results provided genetic and molecular evidence that like its homolog in tobacco, Arabidopsis *NSN1* regulates cell cycling. Therefore, besides its function in embryogenesis and the development of leaf and flower organs [15, 16], the nuclear protein NSN1 is indispensable for maintaining proper cell proliferation in Arabidopsis.

It appears that nucleosome assembly protein AtNAP1;1 and NSN1 may function together as subunits of a functional protein entity. This view is supported by the facts that the two proteins share several common features, including involvement in cell cycle progression, nucleus-localization and the resemblance of their mutants in response to genotoxics. As conserved histone chaperones, plant NAP1s and NRPs have been documented in both monocot and dicot species [18, 19, 22, 23, 26, 38]. In Arabidopsis, for example, overexpression of *AtNAP1;1* increases the expression of *CYCB1;1* and shortenes G2 phase, thereby promoting cell division [24]. The double mutant *nrp1-1 nrp2-1* exhibits arrested cell cycle progression at G2/M [38]. In tobacco, *NAP1* genes have been observed with high expression levels at the G1/S transition [22]. Together with our findings that mutation of *NSN1* caused defect of G1-S and G2-M transition, we concluded that both AtNAP1;1 and NSN1 play roles in the regulation of cell cycle progression.

Supportively, intracellular localization analysis of NSN1 and AtNAP1;1 has shown  that both proteins reside in the nucleus [14, 15, 18]. The result that the N-terminal domain of NSN1 primarily determined its localization [14, 15] is in agreement with the findings of mammalian NS, whose nucleolar localization is specified by the N-terminal basic domain [1]. Based on our findings that the N-terminal domain of NSN1 was essential for its interplay with AtNAP1;1, we postulate that the two proteins interact in the nuclei. This postulation was supported by the BiFC experiment showing in vivo interaction between NSN1 and AtNAP1;1 in the nuclei of tobacco leaves. Interestingly, like NS in mammals [11, 13], AtNAP1;1 is localized in both the nucleus and the cytoplasm, and the protein appears to shuttle between the two organelles [18]. Studies of gain- or loss-of-function mutants have revealed that when localized to the nucleus, *AtNAP1;1* promotes cell division during the early stage of leaf development [24]. The co-localization of NSN1 and AtNAP1;1 in the nucleus (Fig. 6) provides new evidence that both proteins may function as subunits of a complex in cell cycling regulation. Further investigation of the shuttling mechanism of the two proteins will provide valuable information for the functional characterization.

Additionally, the phenotypic features of *Atnap1;1* are highly reminiscent of those of *nsn1* upon subjected to UV or other DNA damage agents. AtNAP1;1 belongs to a multi-gene family. Mutations of *AtNAP1s* individually or simultaneously barely cause visible phenotypes under standard laboratory conditions [23, 26], indicating the dispensability of these genes for plant growth and development under normal conditions. When exposed to bleomysin or UV treatment, *nsn1* and *Atnap1* mutants including single, double, triple and quadruple ones, together with *Atnrp1 and Atnrp2*, exhibit hypersensitivity to the genotoxic agents [18, 26, 38]. The similar response of NSN1 and AtNAP1s to genotoxic treatment suggests their involvement in the DNA-damage repair process although the functional mechanism is unclear.

It has been reported that in mammals NSN1 forms a large protein complex with DDX21, EBNA1 binding protein 2 (EBP2), Pescadillo (PES), and a subset of ribosomal proteins [7]. In Arabidopsis, AtEBP2 and AtPES, the orthologs of EBP2 and PES, respectively, have also been found to interact with NSN1, especially its N-terminal domains (1–174 amino acids) [14], suggesting that NSN1 and its interacting partners regulate plant growth by modulating ribosome biogenesis. In search for potential NSN1-interacting  partners involved in cell cycling regulation, *AtNAP1;1* (At4g26110.2) was identified by the yeast two-hybrid system (Fig. 6). In Arabidopsis, there are four AtNAP1 family members (AtNAP1;1, AtNAP1;2, AtNAP1;3 and AtNAP1;4) and two NRP proteins (AtNRP1 and AtNRP2) [18, 38], clustering in two clades in the phylogenetic tree (Additional file 1: Figure S1a). In AtNAP1 subgroup, AtNAP1;1 shares a sequence identity of 80.3, 72.4 and 46.4% with AtNAP1;3, AtNAP1;2 and AtNAP1;4, respectively (Additional file 2: Table S1). The interaction of AtNAP1;1 with NSN1 might somehow attribute to the divergent C-terminus of AtNAP1;1 (Additional file 1: Figure S1b). Another possible reason why *AtNAP1;1* was singled out from the screening might be that the coverage of the flower library used as pray in this experiment was not sufficient to contain low expression genes such as *AtNAP1;3*, whose expression level in flower is about half of *AtNAP1;1* (Additional file 1: Figure S1c) according to the data from Arabidopsis eFP Browser (http://bar.utoronto.ca/efp_arabidopsis) [39]. Further investigation by deleting the domain(s) of AtNAP1;1 will provide information on the domain(s) of AtNAP1;1 that is required for interaction with NSN1. Future study will focus on the *nsn1 Atnap1;1* double mutant to unveil the molecular functions of *NSN1* and *AtNAP1;1* in regulating plant growth and development as interacting partners.

## Conclusions

The biological functions of Arabidopsis *NSN1* in maintaining proper cell cycle progression were characterized by molecular and genetic approaches. Our results provide direct evidence that the dwarfism of *nsn1* is resulted from the improper cell proliferation in the meristems of both shoot and roots of the mutant plant. As a positive cell cycle regulator, Arabidopsis NSN1 is co-localized and interacts with AtNAP1;1, a nucleosome assembly protein, in the nucleus. The two proteins are involved in regulation of cell cycle progression and their mutants, *nsn1* and *Atnap1;1*, are hypersensitive to treatments of  genotoxic agents. We propose that NSN1 and AtNAP1;1 act together as subunits of a functional protein complex in regulation of cell cycling progression. Hence, like its homologs in mammals, NSN1 in Arabidopsis functions conservatively in regulating plant cell cycling. This study sheds new light on the crosstalk between *NSN1* and *AtNAP1;1* in cell cycle regulation in plants.

## Methods

### Plant materials and growth conditions

The *nsn1-1* T-DNA insertion line (SALK_029201) was obtained from the *Arabidopsis* Biological Resource Center (ABRC) [16], and the *CYCLIN B1::GUS* expressing plant was a gift from Prof. Peter Doerner (University of Edinburg, UK). Plants were grown in soil under normal conditions with 16 h light/8 h darkness at 21 °C.

### Plant treatment

Sterilized Arabidopsis (Col-0 and *nsn1*) seeds were treated at 4 °C for 2 days before moving to normal conditions for germination on half strength MS plates. Plates were placed vertically during seed germination and 4 days after germination, seedlings were transferred to half strength MS plates (control) or half strengh MS plates supplemented with methyl methanesulfonate (MMS) at concentrations of 25, 50, 75 and 100 ppm, or with bleomycin at 3, 6, 9, and 12 mg/L. Images were captured and survival rates were recorded after genotoxic treatment with MMS and bleomycin for one and 3 weeks, respectively.

### Constructs and *Agrobacterium*-mediated transient expression in *N. benthamiana*

For the biomolecule fluoresence complementation (BiFC) experiment, the coding region of *NSN1* cDNA was amplified by RT-PCR using primers pBD-NSN F: 5′-GTC GAC AGA TGG TGA AAC GGA GTA AAA AGA G-3′ and pBD-NSN R: 5′-GTC GAC TTT TTC TTC GGC AAA AGT CCA G-3′, and cloned into vector pCR2.1 (Invitrogen). To express YFP^N^-NSN1, the *NSN1* cDNA fragment was ligated into vector pSPYNE (R) 173 [35] after digestion by S*al I.* For construction of *NAP1;1-cYFP*, the *NAP1;1* (At4g26110.2) coding region was amplified by RT-PCR using primers pNAP1;1 F: 5′-GAA TTC ATG AGC AAC GAC AAG GAT AGC -3′ and pNAP1;1 R: 5′- GTC GAC ACA AAT AAA CTT TAG TTC TGA AAG G-3′. The PCR product was cloned into vector pCR2.1, and then sub-cloned into pSPYCE (M) 155.

For infiltration of tobacco leaf, the two constructs were transformed into *Agrobacterium tumefaciens* GV3101 and infiltration was carried out as described [35].

### Scanning electronic microscopy (SEM) and microscopy

SEM was carried out as described [15]. Briefly, plant tissues were fixed overnight in 50 mM phosphate buffer containing 2% glutaraldehyde and 2% paraformaldehyde. Fixed tissues were rinsed three times with 50 mM phosphate buffer and kept in osmium tetroxide (1%) at 4 °C overnight. After being rinsed three times with 50 mM phosphate buffer, the tissues were dehydrated in a 30, 50, 70, 95 and 100% alcohol gradient. Dehydrated tissues were coated with gold and observed under SEM.

Leaf initiation was defined as described [40]. For measurement of leaf area, the dissected fifth leaf was pictured daily and leaf area was measured using ImageJ. Cell number per leaf was calculated as leaf area divided by cell area. All dissected leaves were treated with chloral hydrate for 3 days. After clearing, photos of palisade cells were taken using Zeiss microscope with DIC lens. For individual leaf, area of 10 palisade cells was measured using ImageJ and an average area per palisade cell was calculated accordingly. For each time point, the fifth leaf from 5 plants was measured and three biological replicates were conducted.

For root length, 70 sterilized Arabidopsis seeds of wild type and *nsn1* were germinated on 1/2 MS medium with 0.8% agar. Root tip was marked for daily image capture and root length was measured by ImageJ. For analysing the cell production per day, 4 days after germination, ten primary root tips grown during the last 24 h were cut and mounted daily on slide using buffer (chloral hydrate: glycerol: water = 8:3:1). DIC optics of a Zeiss confocal microscope (Zeiss Axioskop, Germany) was used for image capture. The number of cortex cells on one side of a root tip was counted.

### Quantitative RT-PCR

Total RNA was isolated from 10-day-old seedlings of wild type and *nsn1* using the RNeasy plant kit followed by treatment with RNase-free DNase I according to the manufacturer instructions (Qiagen, Germantown, MD). RNA of 2 μg was used as template for first strand cDNA synthesis using SuperScrit III (Invitrogen). cDNA was diluted 100 times and 5 μl of cDNA was used as PCR template. Real-time PCR was performed using a mix containing 10 μl of 2× SYBR Premix Ex Taq (Takara Bio Inc., Otsu, Shiga, Japan), 0.8 μl of forward and reverse primer mix (0.2 μM final concentration), 0.4 μl of 50 ROX Reference Dye II, and 3.8 μl of deionized water. PCR was run on an ABI 7500 fast real time PCR system (Applied Biosystems) using a 2-min initial denaturation at 95 °C, followed by 40 cycles of 95 °C (15 s) and 60 °C (40 s). Primers used for qRT-PCR were as follows: for *CYCA2;3* (F: 5′-GGC TAA GAA GCG ACC TGA TG-3′ and R: 5′-TAC AAG CCA CAC CAA GCA AC-3′); for *CYCB1;1* (F: 5′-AAG CTT CCA TTG CAG ACG A-3′ and R: 5′-AGC AGA TTC AGT TCC GGT C-3′); for *CYCD3;1* (F: 5′-ACA ACT CTC GTG CAT TAA CAG GAA-3′ and R: 5′-GAA GAT TGG ATT TGG ATC TGT AAA C-3′); for *H4* (F: 5′-TTA GGC AAA GGA GGA GCA AA-3′ and R: 5′-CTC CTC GCA TGC TCA GTG TA-3′); and for *RNR* (F: 5′- CAA GTG GCT CAG GAC TGT CA-3′ and R: 5′-TCC ATC AGG TCA ACA GCT TG-3′). *Actin2* (*At3g18780*) was used as an internal control (F: 5′-TGG TGT CAT GGT TGG GAT G-3′ and R: 5′-CAC CAC TGA GCA CAA TGT TAC-3′). The relative expression level was calculated based on the value of ΔΔCt.

### Flow cytometry

Flow cytometry was carried out as described [41] with minor modifications. Briefly, leaves (4-days after initiation or fully-expended leaves) were chopped with razor blade into fine strips in cold nuclei isolation buffer (Partec, Müster, Germany). After filtration, the extracts were kept on ice until measurement. The DNA content of nuclei was measured using FACS Caliber flow cytometry (BD Biosciences, USA). Cell nuclei were stained with 2 μg mL^− 1^ DAPI. Each sample was prepared three times and subjected to FACS Caliber cytometry independently. A total of around 10,000 nuclei were measured per analysis.

### Yeast two-hybrid assay

Yeast two-hybrid experiments were carried out as described (Stratagene, USA). ABRC stock CD4-30, an *Arabidopsis* cDNA library of inflorescence meristem, floral meristem and floral buds up to stage 8, was used as pray. The cDNA library was cloned into the E*coR* I – X*ho* I site of pAD-Gal4-2.1 (Stratagene), and transformed into yeast strain PJ69-4a (*his, leu, ura; Gal1-HIS3, Gal2-ADE2, Gal7-LacZ*). The full length *NSN1* coding region and the fragment corresponding to the BC domain were amplified using primers pBD-NSN (F: 5′-GTC GAC AGA TGG TGA AAC GGA GTA AAA AGA G-3′; R: 5′-GTC GAC TTT TTC TTC GGC AAA AGT CCA G -3′) and pBD-BC (F: 5′-GTC GAC AGA TGG TGA AAC GGA GTA AAA AGA G-3′; R: 5′-GTC GAC CTC TTC ATG CTT ATT GGG ACC GGC-3′), respectively. The two fragments were cloned into pBD-GAL4 (Cam) and used as bait after transformation into yeast strain PJ69-4α (*his, leu, ura; Gal1-HIS3, Gal2-ADE2, Gal7-LacZ*). The full-length CDS of *AtNAP1;1* (*At4g26110.2*) cloned into pAD-Gal4-2.1 was amplified with primers (pNAP1;1 F: 5′-GAA TTC ATG AGC AAC GAC AAG GAT AGC-3′; pAD-NAP1;1 R: 5′-CTC GAG AAT AAA CTT TAG TTC TGA AAG -3′). Cells of the two yeast strains were mated and selected on a plate supplemented with Glu-Trp-Leu-Ade. Yeast colonies were further selected for growth on  an Ade Selection Plate (Glc-Ade-Trp-Leu + His). For X-gal overlay assay, agarose solution containing 1 mg/ml 5-bromo-4-chloro-3-indoly-galactopyranoside (X-Gal) in Z buffer was brought to 55 ^o^C and poured to the cooled yeast colonies grown on the Ade selection plates.

### Quantification of Y2H ß-Galactosidase assay

ß-Galactosidase activity was measured using Y2H ß-Galactosidase Kit (Molecular Biotechnology). Briefly, several yeast colonies picked from selection plate were inoculated into 1 ml of the appropriate selective medium and cultured to the late exponential phase. Yeast cells were collected and resuspended in lysis mixture containing Dye solution. Color development was monitored by spectrophotometer at 615 nm. Three independent biological replicates were performed and difference was evaluated by Student’s t test.

## Additional files

Additional file 1: **Figure S1.** Sequence analysis and expression profiles of NAP1 family members. **a**. Homology analysis of AtNAP1 and AtNRP proteins using DNAMAN version 7. Sequence accession number: AtNAP1;1 (AT4G26110.2); AtNAP1;2 (AT2G19480); AtNAP1;3 (AT5G56950); AtNAP1;4 (AT3G13782); AtNRP1 (AT1G74560) and AtNRP2 (AT1G18800). **b**. Sequence alignment of AtNAP1s and AtNRPs. Black represents conserved amino acids (consensus), pink for 75% identity, blue for 50% and yellow for 33% identity. **c**. Comparison of the transcriptional expression pattern of *AtNAP1* paralog genes in flower from Arabidopsis eFP Browser (http://bar.utoronto.ca/efp_arabidopsis). (JPG 3822 kb)

Additional file 2: **Table S1.** Analysis of protein identity among AtNRP1s and AtNRPs. (PDF 98 kb)

## Abbreviations

DAG: Days after germination
DAI: Days after initiation
GUS: β-glucuronidase
MDM2: Murine Double Minute 2
NAP1: NUCLEOSOME ASSEMBLY PROTEINS1
NRPs: NAP1-RELATED PROTEINs
NS: Nucleostemin
NSN: Nucleostemin-like
