# Supplementary figures and images for: Arabidopsis NUCLEOSTEMIN-LIKE 1 (NSN1) regulates cell cycling potentially by cooperating with nucleosome assembly protein AtNAP1;1
Source: BMC Plant Biol. 2018 Jun 1;18:99. doi: 10.1186/s12870-018-1289-2 (PMC5984758; doi:10.1186/s12870-018-1289-2)

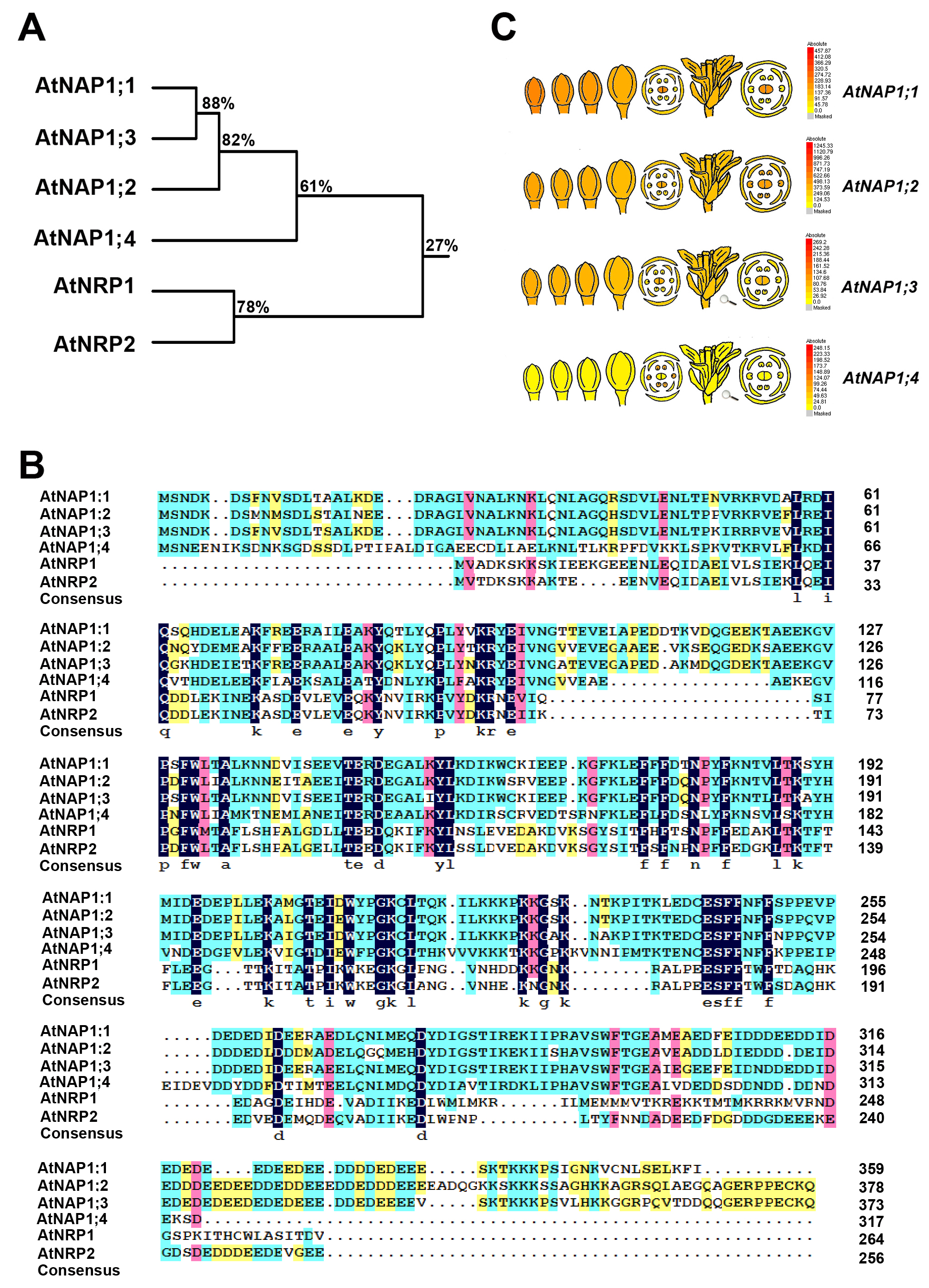

Supplement: Supplementary file 1 — Figure S1. Sequence analysis and expression profiles of NAP1 family members. a. Homology analysis of AtNAP1 and AtNRP proteins using DNAMAN version 7. Sequence accession number: AtNAP1;1 (AT4G26110.2); AtNAP1;2 (AT2G19480); AtNAP1;3 (AT5G56950); AtNAP1;4 (AT3G13782); AtNRP1 (AT1G74560) and AtNRP2 (AT1G18800). b. Sequence alignment of AtNAP1s and AtNRPs. Black represents conserved amino acids (consensus), pink for 75% identity, blue for 50% and yellow for 33% identity. c. Comparison of the transcriptional expression pattern of AtNAP1 paralog genes in flower from Arabidopsis eFP Browser (http://bar.utoronto.ca/efp_arabidopsis). (JPG 3822 kb) [file 12870_2018_1289_MOESM1_ESM.jpg]
