# Supplementary material for: Arabidopsis NUCLEOSTEMIN-LIKE 1 (NSN1) regulates cell cycling potentially by cooperating with nucleosome assembly protein AtNAP1;1
Source: BMC Plant Biol. 2018 Jun 1;18:99. doi: 10.1186/s12870-018-1289-2 (PMC5984758; doi:10.1186/s12870-018-1289-2)
Supplement: Supplementary file 2 — Table S1. Analysis of protein identity among AtNRP1s and AtNRPs. (PDF 98 kb) [file 12870_2018_1289_MOESM2_ESM.pdf]

Table S1. Analysis of protein identity among AtNRP1s and AtNRPs.

	AtNAP1;1	AtNAP1;2	AtNAP1;3	AtNAP1;4	AtNRP1	AtNRP2
AtNAP1;1	100.0%					
AtNAP1;2	72.4%	100.0%				
AtNAP1;3	80.3%	76.8%	100.0%			
AtNAP1;4	46.4%	46.2%	44.9%	100.0%		
AtNRP1	18.9%	17.6%	19.2%	20.4%	100.0%	
AtNRP2	21.9%	22.4%	22.9%	19.6%	71.3%	100.0%
